# Effects of Temperature on Sound Production and Auditory Abilities in the Striped Raphael Catfish *Platydoras armatulus* (Family Doradidae)

**DOI:** 10.1371/journal.pone.0026479

**Published:** 2011-10-17

**Authors:** Sandra Papes, Friedrich Ladich

**Affiliations:** Department of Behavioural Biology, University of Vienna, Vienna, Austria; Claremont Colleges, United States of America

## Abstract

**Background:**

Sound production and hearing sensitivity of ectothermic animals are affected by the ambient temperature. This is the first study investigating the influence of temperature on both sound production and on hearing abilities in a fish species, namely the neotropical Striped Raphael catfish *Platydoras armatulus*.

**Methodology/Principal Findings:**

Doradid catfishes produce stridulation sounds by rubbing the pectoral spines in the shoulder girdle and drumming sounds by an elastic spring mechanism which vibrates the swimbladder. Eight fish were acclimated for at least three weeks to 22°, then to 30° and again to 22°C. Sounds were recorded in distress situations when fish were hand-held. The stridulation sounds became shorter at the higher temperature, whereas pulse number, maximum pulse period and sound pressure level did not change with temperature. The dominant frequency increased when the temperature was raised to 30°C and the minimum pulse period became longer when the temperature decreased again. The fundamental frequency of drumming sounds increased at the higher temperature. Using the auditory evoked potential (AEP) recording technique, the hearing thresholds were tested at six different frequencies from 0.1 to 4 kHz. The temporal resolution was determined by analyzing the minimum resolvable click period (0.3–5 ms). The hearing sensitivity was higher at the higher temperature and differences were more pronounced at higher frequencies. In general, latencies of AEPs in response to single clicks became shorter at the higher temperature, whereas temporal resolution in response to double-clicks did not change.

**Conclusions/Significance:**

These data indicate that sound characteristics as well as hearing abilities are affected by temperatures in fishes. Constraints imposed on hearing sensitivity at different temperatures cannot be compensated even by longer acclimation periods. These changes in sound production and detection suggest that acoustic orientation and communication are affected by temperature changes in the neotropical catfish *P. armatulus*.

## Introduction

Ectothermic animals are dependent on environmental heat sources and control their body temperature through external means. Compared to endothermic animals, they maintain relatively low metabolic rates. In general, the speed of all metabolic processes is influenced by the body temperature, which depends on the ambient temperature [1], [2], [3], [4], [5]. Therefore, ambient temperature affects various physiological processes such as neuronal and muscular activities, including all sensory systems in ectothermic animals [6], [7], [8], [9], [10], [11].

In various climates, fish have to deal with seasonal and diurnal fluctuations in water temperature. Fish either cope with temperature fluctuations or they migrate. Thus, the thermal tolerance range of fish species differs to some degree. Certain physical constraints cannot be compensated for even when animals are acclimated [12], [13], suggesting the presence of an optimum temperature range.

Fish have evolved the largest diversity of sound-producing mechanisms among vertebrates, and sounds are emitted in numerous contexts: e.g. disturbance situations, during courtship, competitive feeding, territorial encounters (for reviews see [14], [15], [16], [17]. Representatives of some catfish families possess two different sound-producing mechanisms [18], [19]. High-frequency stridulation sounds are emitted when pressing ridges of the dorsal process of the pectoral spine against the groove of the pectoral girdle while abducting or adducting pectoral spines [20], [21], [22], [23], [24]. In contrast, vibrations of the swimbladder by sonic muscles result in the emission of low-frequency drumming sounds [15], [18], [25]. In the family Doradidae or thorny catfishes, a thin round bony plate termed elastic spring (‘Springfeder’; [26]) vibrates the swimbladder. The elastic spring is rapidly pulled forward during contractions of sonic muscles which originate at the occipital bone and insert at the elastic spring [19], [27].

Effects of temperature have not been studied in broadband stridulation sounds so far, but have been studied in low-frequency sounds such as drumming sounds. In general, the sound duration and the fundamental frequency increased with rising ambient temperature, whereas the pulse period decreased due to the higher muscle contraction rate (Gobiidae: [28], [29]; Sciaenidae: [30]; Triglidae: [31]; Batrachoididae: [32], [33]. Brawn [34] observed a temperature-dependent increase in the number of sounds produced in the cod *Gadus callarias*.

Fish depend on hearing for analyzing the acoustic scene, for orientation, prey and predator detection and for intraspecific communication [35], [36], [37]. Ambient temperature affects hearing in invertebrates and ectothermic vertebrates. Such effects have been examined in insects [38], [39], [40], amphibians [41], [42], [43] and reptiles [44], [45]. In general, raising the temperature increased both the most sensitive (best) frequency and the absolute sensitivity [46], [47]. The number of action potentials increased and the temporal tuning of auditory neurons shifted to higher rates of amplitude modulation [48]. Similar results have been found in the tuning of the auditory system in cicadas and locusts [38], [40].

In fish, only a few studies investigated the effects of temperature changes. Dudok van Heel [49] found that the European minnow, *Phoxinus phoxinus*, can discriminate between higher frequencies at higher ambient temperature. In goldfish, *Carassius auratus*, warming increased the spontaneous activity and sensivity of auditory neurons, the best frequency at a given signal level and the responsiveness to an acoustic stimulus [50]. The walleye pollock, *Theragra chalcogramma*, showed a reduced auditory sensitivity at lower ambient temperature within hours [51]. Wysocki et al. [13] showed that the eurythermic channel catfish, *Ictalurus punctatus,* and the stenothermic tropical catfish *Pimelodus pictus* exhibited higher hearing sensitivity at higher temperatures, especially at the highest frequency tested. Differences between temperatures were more pronounced in the eurythermic catfish species.

Sound characteristics are important for coding information in agonistic and reproductive contexts (conflict resolution, distress situations, courtship, establishment of territories). Fish often produce series of short broad-band pulses, for example in the stridulation sounds of catfishes and gouramis [18], [52], with distinct temporal patterns and variable interpulse intervals [52], [53]. Severals studies suggest that temporal patterns are important carriers of information in fish [53], [54]. Wysocki and Ladich [54] showed that the auditory system of the catfish *Platydoras armatulus* (formerly *P. costatus*) and the croaking gourami *Trichopsis vittata* were able to process each pulse within a stridulation sound.

The present study was designed to investigate the effects of temperature on (1) sound production and sound characteristics, (2) the absolute auditory sensitivity and (3) the ability of the auditory system to resolve temporal patterns of sounds in the Striped Raphael catfish.

The neotropical catfish *P. armatulus* [55] was chosen because this group produces two different sound types (swimbladder and pectoral stridulatory sounds) and because it possesses accessory hearing structures (Weberian apparatus). Groups with accessory hearing structures that couple air-filled cavities acoustically to the inner ear are most likely affected by temperature changes as shown previously [13], [56]. *Platydoras armatulus* inhabits the Amazonian river system and is known to emit both types of sounds in distress situations [18]. This is the first study in which the effects of temperature on both vocalization and hearing have been examined in the same fish species.

## Results

### Stridulation sounds

**Figure 1:**
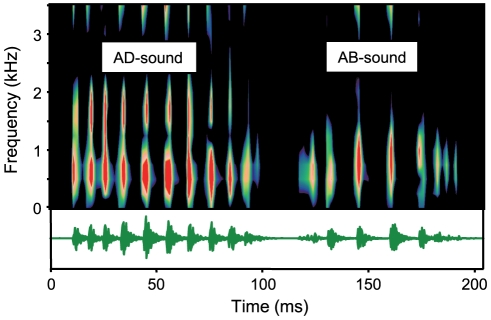
Sonagram and oscillogram of stridulation sounds. Stridulation sounds can be produced during an adduction (AD) and abduction (AB) movement of pectoral fins. The main energies of these broadband sound ranged from 0.3 to 1.4 kHz. Sampling rate 44.1 kHz, filter bandwidth 320 Hz, hanning filter, overlap 30 %.

All *P. armatulus* produced sounds by moving the pectoral fins forward (abduction, AB) and backward (adduction, AD), utilizing either one or both fins at the same time. Fish could also move fins without emitting sounds or lock spines in an abducted position. Subjects usually started producing sounds with an adduction movement because they spread their pectoral fins in an adducted position during handling. Stridulation sounds consisted of series of broadband pulses with main energies ranging from 0.3 to 1.3 kHz (Fig. 1). All fish emitted stridulation sounds when hand-held (but not all produced drumming sounds).

**Figure 2:**
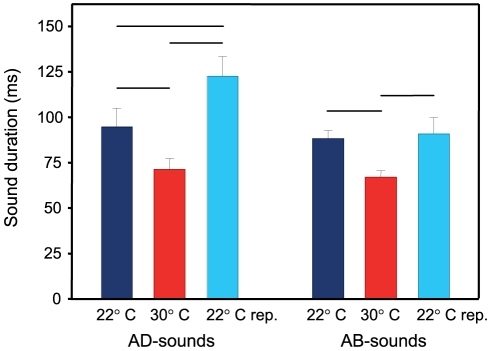
Sound duration of stridulation sounds at 22 and 30°C. Mean (±S.E.) duration of AD- and AB-stridulation sounds in *P. armatulus* kept at 22°C, 30°C and 22°C repeated (rep.). N = 8 fish per temperature. Horizontal bars indicate significant differences between temperatures (p≤0.05).

In AD- and AB-stridulation sounds, sound duration showed significant differences between temperatures (AD-stridulation sounds: Friedman-test, χ^2^ = 14.250, df = 2, p≤0.01; AB-stridulation sounds: Friedman-test, χ^2^ = 10.750, df = 2, p<0.01). In both sound types, duration was significantly shorter at 30°C (Wilcoxon-tests for AD: 22°C versus 30°C: Z = 2.38, p<0.02; 30°C versus 22°C repeated: Z = 2.52, p<0.02; 22°C versus 22°C repeated: Z = 2.52, p<0.02; Wilcoxon-tests for AB-stridulation sounds: 22°C versus 30°C repeated: Z = −2.52, p<0.02; 30°C versus 22°C repeated: Z = −2.38, p<0.02; 22°C versus 22°C repeated: Z = −0.42, p>0.05) (Fig. 2) (Tab. 1). No temperature-dependent differences were found in the number of pulses in either type (AD-stridulation sounds: Friedman-test, χ^2^ = 2.250, df = 2, p>0.05; AB-stridulation sounds: Friedman-test, χ^2^ = 1.067, df = 2, p>0.05) (Tab. 1).

**Table 1:** Sound characteristics in stridulation sounds at the experimental temperatures.

Temperature	22°C		30°C		22°C repeated	
	AD	AB	AD	AB	AD	AB
Duration (ms)	94.8±10.0	88.4±4.4	71.5±5.9	67.1±3.6	122.7±10.8	91.0±8.8
Number of pulses	7.9±1.0	7.6±0.3	6.0±0.6	7.8±1.1	7.7±1.2	6.2±0.9
Minimum pulse period (ms)	7.7±1.3	5.8±0.6	7.4±1.2	5.1±0.7	8.8±1.3	7.7±0.7
Maximum pulse period (ms)	23.2±4.9	18.0±1.2	20.7±3.0	17.2±2.3	29.2±4.8	26.1±4.4
SPL (dB re 1 µPa)	136.4±0.7		137.9±1.0		136.6±1.1	
Dominant frequency (Hz)	601.6±118.9		1271.9±107.5		1203.0±133.1	

Mean (±SE) sound duration, number of pulses, minimum and maximum pulse period, sound pressure level (SPL) and dominant frequency in AD- and AB-stridulation sounds in *P. armatulus*. N = 8.

The pulse period showed great variability among and within individuals. In general, the periods were longest in the centre of the stridulation sounds and became shorter at the beginning and at the end of the stridulation sounds (Fig. 1, see Material and Methods). The mean minimum pulse period ranged from 7.4–8.8 ms in AD- and from 5.1–7.7 ms in AB-stridulation sounds (Tab. 1). A Friedman-test (χ^2^ = 7.40, df = 2, p<0.05) followed by a Wilcoxon-test revealed that the minimum pulse periods in AB-stridulation sounds were significant shorter at 30°C than at 22°C repeated (Z = −2.521, p<0.05). The minimum pulse periods of AD-stridulation sounds and maximum pulse periods of AD- or AB-stridulation sounds did not change with temperature.

Sound pressure levels did not change significantly with temperature and remained almost constant at about 137 dB rel 1 µPa ( Friedman-test, χ^2^ = 2.250, df = 2, p>0.05) (Tab. 1). Otherwise, the dominant frequency revealed significant differences between 22°C and 30°C and between 22°C and 22°C repeated (Wilcoxon-test, Z = −2.380, p≤0.05). Dominant frequency doubled after fish were acclimated to 30°C from 601.6 Hz to 1271.9 Hz, but did not decrease when repeating the 22°C measurements.

### Drumming sounds

**Figure 3:**
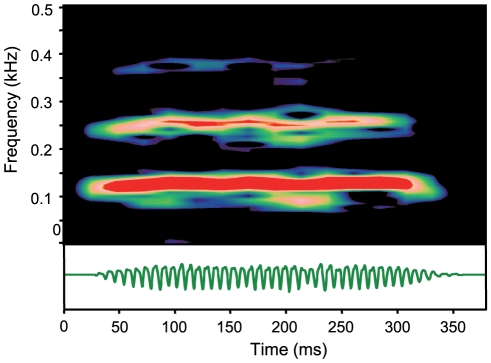
Sonagram and oscillogram of a long drumming sound. The sonagram shows three harmonics with the main energy concentrated at the first harmonic between 100 and 150 Hz (fundamental frequency). Sampling rate 44.1 kHz, Filter bandwidth 10 Hz, hanning filter, overlap 75 %.

*P. armatulus* emitted two different types of drumming sounds: series of short drumming sounds and single long drumming sounds. Series of short drumming sounds were recorded in 6 out of 8 animals but not at all temperatures (22°C: N = 4; 30°C: N = 4; 22°C repeated: N = 1). Long drumming sounds, in contrast, were recorded in every individual but again not at every temperature (22°C: N = 5; 30°C: N = 8; 22°C: repeated N = 5). The long drumming sounds revealed a harmonic structure with fundamental frequencies (drumming muscle contraction rate) between 100 and 150 Hz (Fig. 3).

*P. armatulus* produced more stridulation than drumming sounds. Stridulation sounds were produced by each individual at both temperatures which was not the case in drumming sounds. Stridulation sounds and drumming sounds were often emitted simultaneously. In general, long drumming sounds were longer than stridulation sounds, in some cases over 300 ms. Long drumming sound duration did not change significantly with temperature (Tab. 2) (Kruskal-Wallis test, χ^2^ = 1.411, df = 2, p>0.05). Accordingly, the mean number of pulses in drumming sounds did not change either (Kruskal-Wallis test, χ^2^ = 3.740, df = 2, p>0.05 ).

**Table 2:** Sound characteristics of long drumming sounds at the experimental temperatures.

Temperature	22°C	30°C	22°C repeated
Duration (ms)	277.5±100.7	277.2±41.0	326.6±65.4
Number of pulses	16.7±5.7	27.9±4.7	25.2±5.1
Mean pulse period (ms)	14.4±0.4	10.4±0.8	12.8±0.6
Fundamental frequency (Hz)	74.2±2.4	99.1±7.9	75.5±1.5

Mean (±SE) sound duration, number of pulses, pulse period and fundamental frequency in drumming sounds in *P. armatulus*. N = 5 kept at 22°C and 22°C repeated; N = 8 at 30°C.

**Figure 4:**
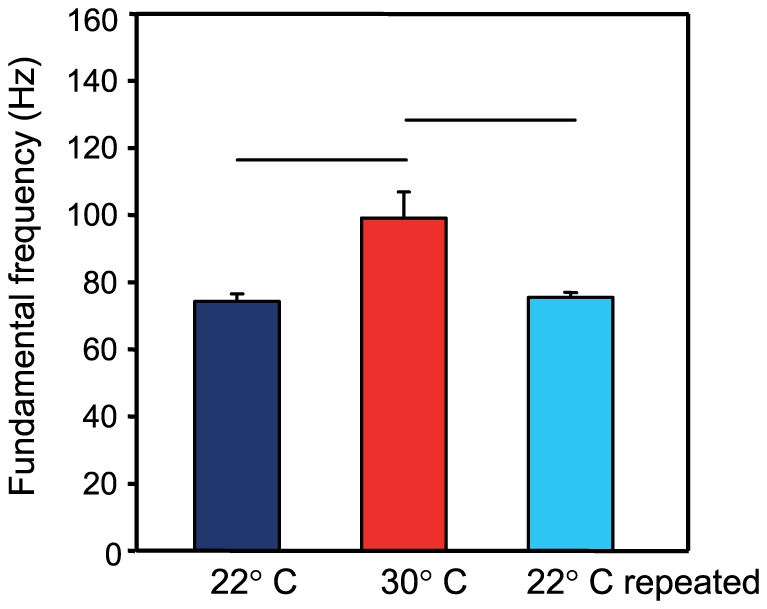
Fundamental frequency of drumming sounds at 22 and 30°C. Mean (±S.E.) fundamental frequency of long drumming sounds in catfishes kept at 22°C, 30°C and 22°C repeated; N = 5 at 22°C and 22°C repeated; N = 8 at 30°C. Horizontal bars indicate significant differences between temperatures (p≤0.05).

The fundamental frequency in drumming sounds differed significantly between temperatures (Kruskal-Wallis test: χ^2^ = 10.05, df = 2, p<0.01; Fig. 4). Bonferroni-corrected posthoc tests revealed that the fundamental frequency was significantly lower at the lower temperature (22°C versus 30°C: U-test, U = 2.0, N_1_ = 5, N_2_ = 8, p<0.01; 30°C versus 22°C repeated: U = 2.5, N_2_ = 8, N_3_ = 5, p≤0.01; 22°C versus 22°C repeated: U-test; U = 10.5, N_1_ = 5, N_3_ = 5, n.s.) (Tab. 2). Pulse periods in drumming sounds differed significantly between temperatures (Kruskal-Wallis test: χ^2^ = 10.50, df = 2, p<0.01). Bonferroni-corrected posthoc tests revealed that the pulse period decreased significantly when the temperature raised from 22°C to 30°C (U-test, U = 1.0, N_1_ = 5, N_2_ = 8, p<0.005). No differences were found between 30°C and 22°C repeated and 22°C and 22°C repeated.

### Auditory abilities

**Figure 5:**
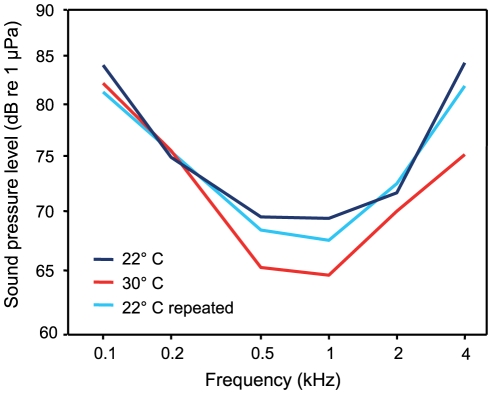
Auditory evoked potential audiograms at 22°C and 30°C. Mean hearing thresholds of *P. armatulus* kept at 22°C, 30°C and 22°C repeated. N = 8 per temperature.

Best hearing occurred at 0.5 and 1 kHz at both temperatures (Tab. 3, Fig. 5). A two-factorial ANOVA revealed that the auditory sensitivity differed between temperatures (F_2,126_ = 13.46, p<0.001) and that there was a significant interaction between temperature and frequency (F_10,126_ = 2.15, p≤0.05). Thus, changes in auditory sensitivity showed different trends at different frequencies. The hearing sensitivity was higher at the higher temperature and differences were more pronounced at higher frequencies (0.5–4 kHz).

**Table 3:** Hearing threshold values at the experimental temperatures.

Frequency (kHz)	22°C	30°C	22°C repeated
0.1	82.0±0.8	82.1±1.1	84.0±1.1
0.2	76.1±1.2	75.5±1.6	74.9±0.7
0.5	69.0±1.5	65.3±1.6	69.5±0.8
1	68.1±1.0	64.6±1.5	69.4±1.0
2	73.1±1.5	70.0±1.3	71.6±1.1
4	82.6±1.6	75.1±1.6	84.3±1.3

Mean (±S.E.) hearing thresholds of *P. armatulus* kept at 22°C, 30°C and 22°C repeated. N = 8.

A Bonferroni Post-hoc test showed a significant difference between the 30°C and both 22°C audiograms (22°C versus 30°C: p≤0.001; 30°C versus 22°C repeated: p≤0.001; 22°C versus 22°C repeated: p≤0.001).

### Waveforms and latencies in response to single clicks

**Figure 6:**
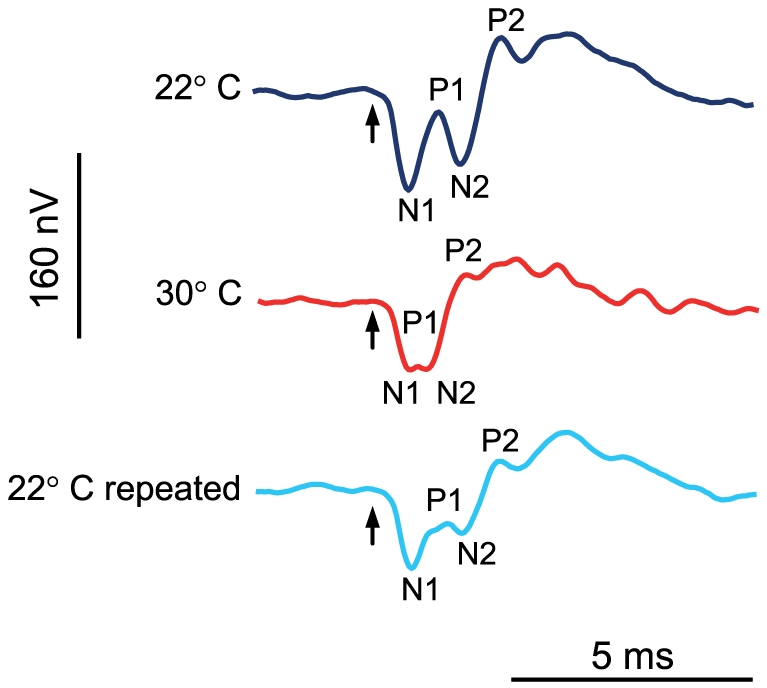
AEPs of one specimen of *P. armatulus* in response to a single-click stimulus. Click stimulus was presented at 28 dB above hearing thresholds at both temperatures. Arrows indicate the onset of the single-click stimulus.

AEPs of *P. armatulus* in response to clicks consisted of a series of negative and positive deflections whose amplitude decreased when lowering the SPL. AEPs started with a negative peak (Fig. 6). The most constant peaks – N1, P1, N2 and P2 – occurred in the AEPs in response to a single-click presentation at 22°C and 30°C. Significant differences in latencies of peaks P1, N2 and P2 were found between temperatures (P1: Friedman-test, χ^2^ = 12.0, df = 2, p<0.01; N2: Friedman-test, χ^2^ = 13.231, df = 2, p<0.01; P2: Friedman-test, χ^2^ = 12.250 , df = 2, p<0.01). The delay in the onset of P2 was significantly longer at lower temperature (Tab. 4) (22°C and 30°C: Wilcoxon-test, N = 8, p≤0.05; 30°C and 22°C repeated: Wilcoxon-test, N = 8, p≤0.05). The peak-to-peak amplitude between the first positive peak and the second positive peak increased with rising temperature. N1 and N2 tended to fuse at higher temperature, whereas P1 almost disappeared (Fig. 6).

**Table 4:** Latencies of single-clicks measured at the experimental temperatures.

Peak	22°C	30°C	22°C repeated
N1	0.99±0.02	1.04±0.08	1.03±0.03
P1	1.49±0.04	1.16±0.02	1.66±0.03
N2	2.03±0.04	1.40±0.03	2.14±0.04
P2	2.81±0.06	2.38±0.10	2.96±0.04

Mean (±S.E.) latency (ms) of negative peaks (N1, N2) and positive peaks (P1, P2) of *P. armatulus* kept at 22°C, 30°C and 22°C repeated calculated as the time period between the onset of a single click stimulus 32 dB above hearing threshold and the peaks. N = 8, except N2 at 30°C (N = 7) and P1 at 30°C (N = 6).

### Temporal resolution measurements

**Figure 7:**
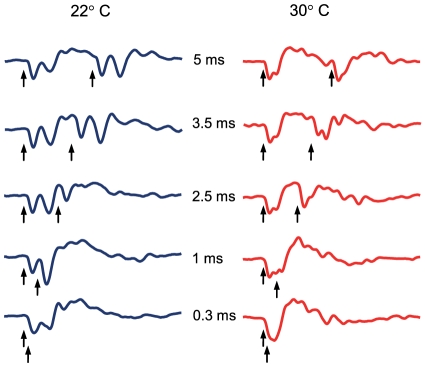
Temporal resolution of double-click stimuli with variable click periods (0.3–5 ms) at 22 and 30°C. AEPs of one specimen of *P. armatulus* in response to a double-click stimulus 28 dB above hearing threshold at different click periods (ms) and temperatures. The arrows indicate the onset of stimuli.

Two distinct AEPs were detectable in response to double-clicks at click periods of 5 ms down to 1.5 ms (Fig. 7). At shorter click periods, the responses to the first and to the second click were partly overlaid (Fig. 7). The minimum resolvable click period was 0.81 ms. Near to the hearing threshold, N1 and N2 as well as P2 and P3 tended to merge until one negative and positive peak remained. AEP shape and latency varied within and between individuals. No significant difference was observed in the minimum resolvable click periods between temperatures (Friedman test: χ^2^ = 3.5, df = 2, p>0.05). Mean minimum gap width ranged from 0.81 (±0.09 SE) to 1.00 ms.

## Discussion

Physiological processes depend on the surrounding temperature in ectothermic animals. This leads to the assumption that both sound production (sound characteristics) and sound detection are affected by the temperature in fishes. Previous studies reveal that, in several vocalizing species, temperature change induced changes in temporal characteristics of sounds including sound duration, dominant/fundamental frequency, and/or sound pressure level [28], [29], [30], [31], [32], [33], [57]. In addition a few studies showed that temperature also affects hearing [13], [51]. However, the present study is the first one investigating such effects on sound communication by studying sound characteristics and hearing abilities in parallel in the same species.

### Temperature effects on sound characteristics

In general, sound duration and fundamental or dominant frequency increased, whereas pulse period and pulse duration decreased with rising ambient temperature. Note, however, that not all sound characteristics are effected by temperature changes in species studied and that opposite trends have been observed in a few cases.

The duration of stridulation sounds in *P. armatulus* was affected significantly at elevated ambient temperature. Both AB- and AD-stridulation sounds became significantly shorter at the higher temperature. This is probably because pectoral muscles contract faster, taking less time for a complete pectoral fin sweep [19].

Stridulation sounds were influenced by temperature, whereas duration of drumming sounds did not change in the current study. Similarly, in the searobin *Prionotus carolinus*, Connaughton [58] reported no relation between sound duration and temperature variation.

Temperature effects on drumming sounds are a well-studied topic in fish biology. Drumming sounds in piranhas, *Serrasalmus nattereri*, in the oyster toadfish, *Opsanus tau*, and in the gobies *Padogobius bonelli* and *P. nigricans* became shorter at higher temperatures [28], [29], [33], [57]. In contrast, drumming sound duration in the weakfish, *Cynoscion regalis*, and in the Lusitanian toadfish, *Halobatrachus didactylus*, increased with rising ambient temperature [30], [32]. Thus, results on sound duration influenced by temperature showed different trends. For instance Amorim [31] reported that in *H. didactylus* ‘knocks became shorter and ‘grunts’ became longer at higher temperature. So far, sound characteristics are temperature-dependent, although no conclusions could be drawn about which factors are responsible for sound lengths either increasing or decreasing with temperature change.

The maximum and minimum pulse periods of stridulation sounds showed temperature-dependence to some degree. The minimum period became shorter in AB-stridulation sounds at higher temperature, and a significant difference was also found between the two cold measurements, whereas in AD-stridulation sounds no trend was detected. The shorter pulse periods at higher temperatures most likely decreased the duration of AB-stridulation sounds because the number of pulses was constant. The lack of such a relationship in AD-stridulation sounds is probably because the minimum and maximum pulse periods do not reflect the mean pulse period of sounds completely. Dominant frequency of stridulation sounds tended to increase with temperature. No comparable studies have been conducted on the temperature-effects on stridulation sound characteristics.

In drumming sounds of *P. armatulus*, the mean pulse period tended to decrease with increasing temperature. The fundamental frequency which reflects the muscle contraction rate increased from approximately 75 Hz to 100 Hz. Drumming muscles are fast-contracting muscles consisting of many thin myofibrils encircled by layers of sarcotubules [27]. A temperature change may affect the pulse pattern generator circuits and the muscle contraction properties that change the contraction rate of the drumming muscles. A warmer sarcoplasmic reticulum can cycle calcium more rapidly in the oyster toadfish *Opsanus tau* [28], [32], [59]. Studies on the Arno goby, *Padogobius nigricans*, the searobin *Prionotus carolinus* and the oyster toadfish, *Opsanus tau,* reported a rise in fundamental frequencies with higher temperature [29], [33], [58]. These studies did not investigate if, due to this outcome, pulse periods decreased with elevated temperature. Interestingly, Connaughton et al. [30] described shorter pulse duration but increasing pulse periods in the weakfish at higher temperature. Nevertheless, sound characteristics such as pulse period and fundamental and/or dominant frequency showed an overall strong correlation with ambient temperature.

In *P. armatulus*, no temperature effect was found on the sound pressure level in stridulation sounds. Those levels ranged from 136.4 to 137.9 dB. Connaughton [58] observed that the sound pressure level of the searobin *Prionotus carolinus* was not influenced by temperature as well. In contrast, lower sound pressure levels have been described in the piranha and the weakfish at lower temperatures [30], [57].

### Temperature effects on hearing

In several ectothermic animals, temperature-dependent effects on the auditory system have been reported. Amphibians showed lower hearing thresholds at higher surrounding temperature [46], [47]. In insects, warming above ambient temperature increased the characteristic hearing frequency or best frequency, the spike rate and the sensitivity [38], [40].

**Figure 8:**
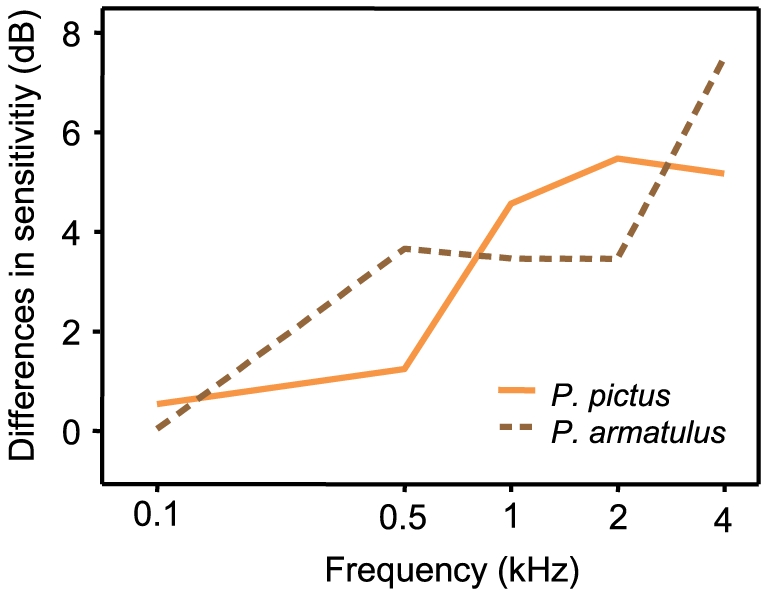
Comparison of the change in hearing sensitivity in the Amazonian catfishes *Pimelodus pictus* (Wysocki et al., 2009) and *P. armatulus* (current study). Differences are shown in both species after acclimation for at least 3 weeks to either 22°C or 30°C.

Higher temperatures induced a frequency-dependent change in sensitivity in all fish species investigated so far [13], [51]. Dudok van Heel [49] was the first to describe temperature effects on the auditory function in fishes. He trained blinded European minnows (*Phoxinus phoxinus*) to react to different frequencies. At higher temperature, the upper limit of frequency discrimination shifted from 1200 Hz up to 1600 Hz. Subsequently, the detectable frequency range became wider. Wysocki et al. [13] were interested if ambient temperature influenced auditory sensitivity in a erythermal and stenothermal catfish differently. Hearing thresholds of the stenothermic tropical catfish *Pimelodus pictus* decreased from 22 to 30°C [13]. *Pimelodus pictus* and *P. armatulus* showed a similar frequency-dependent increase in sensitivity when increasing the ambient temperature by 8°C (Fig. 8).

The eurythermal North American channel catfish *Ictalurus punctatus* differed considerably from the stenothermal tropical catfishes (*P. pictus* and *P. armatulus*) ([13], and current study). The channel catfish exhibited higher changes in hearing sensitivity when the temperature changed, especially at the highest frequency tested. In *I. punctatus*, hearing sensitivity at 4 kHz increased by 23 dB when temperature was raised from 18 to 26°C. Hearing thresholds of the tropical catfish *P. pictus* showed smaller differences (maximum change: 5 dB) at a similar temperature change of 8°C.

Several factors explain the phenomenon that hearing sensitivity at higher frequencies is more affected by temperature changes than at lower frequencies. Fay and Ream [50] concluded that temperature-dependent effects on the nervous system in goldfish, *Carassius auratus*, may reflect changes in the release and reuptake of neurotransmitter at the synapses between hair cells and auditory nerve fibres. Elevated temperature increased the cells' spontaneous activity, sensitivity, best frequency and responsiveness. Wysocki et al. [13] argued that high-frequency hearing needs faster firing of action potentials due to synchronization with the shorter sound cycles. The refractory periods and transduction processes are perhaps more temperature-dependent than those of longer cycles of lower frequencies. This would be consistent with the frequency-dependent improvement of hearing in the present study.

Latencies decreased in three out of four peaks (P1, N2 and P2) at higher temperatures in *P. armatulus*. This result might be explained by temperature dependence of spike conduction velocity, of spike shape and perhaps of synaptic delay. Short latencies indicate better hearing cability at higher temperature [56]. Besides, Wysocki and Popper [60] also observed different AEP shapes at different temperatures. At higher temperature, peaks tended to fuse, especially the first and the second negative peak, and AEP amplitude increased.

In the locust *Locusta migratoria*, higher temperatures resulted in a better resolution of gaps [39]. No such change with temperature was found in the current study. Wysocki and Ladich [54] reported that the mean minimum resolvable pulse period of the Lined Raphael catfish was 0.52 ms, measured at 25°C. The current study found a mean value of 0.86 (±0.05) ms at 32 dB above hearing threshold at both temperatures investigated; two distinct AEPs were clearly traceable at a click period exceeding 3.5 ms (according to [54]). The minimum pulse periods in the stridulation sounds (2 ms) and in the drumming sounds (6 ms) in *P. armatulus* as measured in the recent study are longer than the minimum resolvable click period. This indicates that catfishes encode the temporal information of sounds from conspecifics, independent of changes in ambient temperature.

### Temperature and acoustic communication

Many catfish species produce sounds in various behavioural contexts such as disturbance, agonistic behavior and male courtship display [19], [61], [62]. Thus, the detection of stridulation and drumming sounds is an important factor in catfish behavior. In disturbance situations, catfish are likely to emit more stridulation sounds, whereas in intraspecific contexts more drumming sounds are produced [62]. Accordingly, stridulation sounds may have a warning or defense intention, while drumming sounds play an important role in intraspecific communication [62], [63].

Temperature affects sound characteristics in both stridulation sounds (duration) and drumming sounds (pulse period, fundamental frequency). Both observations agree with the fact that the muscle contraction rate increases with temperature. Higher contraction speed of the pectoral abductor and adductor muscle results in shorter AB- and AD-stridulation sounds. Similarly, a higher drumming muscle contraction rate results in shorter pulse periods and a higher fundamental frequency. Stridulation sounds tended to have higher dominant frequencies and shorter pulse periods. Sound frequencies of both sound types shift to higher frequencies with rising temperatures, and hearing sensitivity increased at higher frequencies. Thus, low-frequency (0.1 and 0.2 kHz) drumming sounds and in particular high-frequency stridulation sounds (above 500 Hz) will be better detectable at higher temperatures. The lower hearing thresholds, together with the faster response of the auditory system (shorter latencies of AEP waves), leads to the assumption that changes in temporal patterns of both types of sounds (duration, pulse periods) are detected and that acoustic communication is facilitated at higher temperatures in catfishes. The habitat temperature typically ranges between 23°and 30°C. Studies on vocalizing species are required to determine whether this effect is more pronounced in eurythermic than stenothermic fish species.

## Materials and Methods

### Ethics Statement

The study protocol was approved by the Austrian Federal Ministry of Science and Research, permit number GZ 66.006/0023-II/10b/2008.

### Animals

Lined Raphael catfish [55] were kept in a community tank (110×55×30 cm, 25±1°C) and a total of 8 adult specimens of *P. armatulus* were used in the present study. They were obtained from a local pet supplier. Groups of four fish were introduced into two experimental tanks (70×40×30 cm) which were equipped with half flower pots and whose bottom was covered with sand. The water was filtered by external filters and a 12∶12 hour light-dark cycle was maintained. Fish were fed with frozen chironomid larvae and flake food five days per week. The size of fish was as follows: total length: 126.2–142.5 mm; standard length: 108.6–121.1 mm; body mass: 27.9–41.8 g. The sex of the fish was not determined because this was not possible without killing the animals.

Temperature in the experimental tanks was changed using submersible heaters by approximately one degree per day until final temperatures of 22±1°C and 30±1°C, respectively, were achieved. Fish were acclimated for at least three weeks to each experimental temperature, first to 22°C, then to 30°C and finally to 22°C again. Auditory measurements were conducted between 24 h and 4 weeks prior to sound recordings. Fish recovered completely within one day.

### Sound and video recordings

Sound and video recordings were conducted in a sound-proof room in a separate recording tank (50×27×30 cm) either at 22±1°C or at 30±1°C, depending on the acclimation temperature in the experimental tank. Fish were hand-held at a distance of 5 to 10 cm from the hydrophone which was positioned in the middle of the recording tank. In order to avoid overlap of stridulation sounds generated simultaneously by both pectoral fins, one fin was fixed.

Sounds and fin movements were recorded using a hydrophone (Brüel & Kjaer 8101) connected to a power supply (Brüel & Kjaer 2804) and an amplifier (AKG B29L), and a video camera (Sony VX1). Both acoustic and video signals were recorded simultaneously on a harddisk video recorder (Panasonic DMR-EX95V). Videorecordings were necessary to determine which sounds were produced during abduction and adduction of pectoral fins.

Sound pressure levels (RMS fast, L-weighting) were recorded using a sound level meter (Brüel & Kjaer Mediator 2238) which was connected to the power supply of the hydrophone. Three walls of the recording tank were lined on the inside by acoustically absorbent material (airfilled packing foil) and its bottom was covered with fine sand. The recording tank supporting table was placed on a vibration-isolating concrete plate.

### Sound analysis

**Figure 9:**
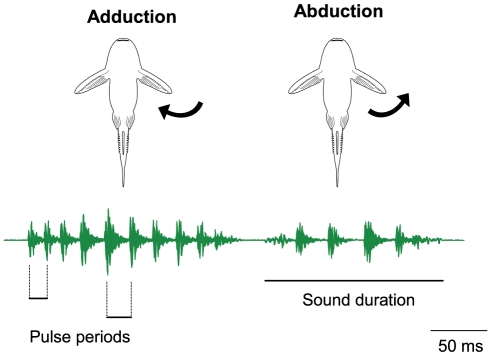
Drawings of the ventral side of the catfish and oscillogram of an AD- and AB-stridulation sound. The upper drawings illustrate the fin movement during production of AB- and AD-sounds, the lower oscillogram shows temporal sound characteristics measured. Sound duration was measured from the beginning to the end of a sound. The pulse period was defined as the time between the peak amplitudes of two subsequent pulses within a sound. A minimum and a maximum pulse period are shown within a stridulatory sound.

Sounds were analysed using Cool Edit 2000 (Syntrillium Software Corporation, Phoenix, USA) and ST^x^ Soundtools 3.7.8. (Institute of Sound Research at the Austrian Academy of Sciences). *P. armatulus* produced sounds during the adduction (AD) and abduction (AB) of pectoral fins [18]. The following sound characteristics were determined in stridulatory sounds: the sound duration (ms), the number of pulses, the minimum and maximum pulse period (ms), the dominant frequency (Hz) and the sound pressure level (dB re 1 µPa) (Fig. 9). In each individual, five AD- and five AB-stridulation sounds (a total of 10 sounds) were examined. In the drumming sounds, the sound duration (ms), the number of pulses, the mean pulse period (ms) and the fundamental frequency (Hz) were determined. Sound pressure levels could not be determined for AB- and AD- stridulation sounds separately because the sound level meter does not allow SPL readings at such short intervalls. Furthermore, SPLs could not be determined for drumming sounds because fish produced stridulation sounds, which were much louder, at the same time.

The pulse period was defined as time between the peak amplitudes of two subsequent pulses within a sound. In stridulation sounds, only sounds consisting of at least four pulses were used for pulse period measurements. The average of the minimum and maximum pulse periods of stridulation sounds (each N = 3) were calculated separately for each fish instead of a total mean due to the large variabilty in these sound characteristics. For each individual, 60 pulse periods were measured at each temperature. The dominant frequencies of stridulation sounds were measured using cepstrum-smoothed power spectra (filter bandwidth 1 Hz, 50% overlap, number of coefficients 100, hamming filter), determined from five AD- and five AB-stridulation sounds, thus 10 stridulatory sounds per fish. A sound file made up of stridulation sounds was created separately to determine individual-specific dominant frequencies.

In drumming sounds, pulse periods were defined as the time between subsequent drumming muscle contractions. Pulse periods were analyzed in at least four drumming sounds per fish (10 pulse periods per fish). The mean pulse period was calculated for each fish. The fundamental frequency of drumming sounds was determined from sound power spectra calculated from 10 sounds per fish. Again, a sound file consisting of drumming sounds of one specimen was created to calculate the fundamental frequency of each individual.

### Auditory sensitivity measurements

Auditory sensitivity was measured using the auditory evoked potential (AEP) recording technique described by Kenyon et al. [64] and modified by Wysocki and Ladich [54], [65]. Test subjects were secured in a round plastic tub (35 cm diameter, 15 cm height, lined on the inside by acoustically absorbent material, 1 cm layer of fine sand) filled with water and adjusted so that the nape of the head was just above the surface of the water, and a respiration pipette was inserted into the animal's mouth. The water temperature was either at 22±1°C or 30±1°C, depending on the temperature in the holding tanks.

Respiration was achieved by a temperature-controlled gravity-fed water circulation system. To immobilize animals and to reduce the myogenic noise level, they were injected with a curariform agent (Flaxedil; gallamine triethiodide; Sigma-Aldrich, Vienna, Austria). The dosage required was 1.5–2.8 µg g^−1^ and allowed the fish to perform opercular movements during the experiment. The plastic tub was positioned on an air table (TCM Micro-g 63–540) which rested on a vibration-isolating concrete plate. The entire setup was enclosed in a walk-in soundproof room which was constructed as a Faraday cage (interior dimensions: 3.2×3.2×2.4 m).

The AEPs were recorded using silver wire electrodes (0.32 mm diameter) that were pressed firmly against the skin, which was covered by small pieces of tissue paper to keep it moist, in order to ensure proper contact during experiments. The recording electrode was placed in the midline of the skull over the region of the medulla and the reference electrode cranially between the nares. Shielded electrode leads were attached to the differential input of an a.c. preamplifier (Grass P-55, Grass Instruments, West Warwick, RI, USA; gain 100x, high-pass at 30 Hz, low-pass at 1 kHz). A ground electrode was placed in the water near the subject. Both stimuli presentation and AEP-waveform recording were accomplished using a Tucker-Davis Technologies (TDT, Gainesville, FL, USA) modular rackmount system (TDT System 3) controlled by a Pentium PC containing a TDT digital processing board and running TDT BioSig RP Software.

### Presentation of sound stimuli

Sound stimuli waveforms were generated using TDT SigGen RP software and fed through a power amplifier (Alesis RA 300, Alesis Corporation, Los Angeles, CA, USA). A dual-cone speaker (Tannoy System 600, frequency response 50 Hz to 15 kHz±3 dB), mounted 1 m above test subjects in the air, was used to present the stimuli during testing. Sound stimuli consisted of tone bursts presented at a repetition rate of 21 s^−1^. Hearing thresholds were determined at frequencies of 0.1, 0.2, 0.5, 1, 2 and 4 kHz, presented in random order. Rise and fall times were one cycle at 0.1 and 0.2 kHz and two cycles at all other frequencies. All bursts were gated using a Blackman window.

The stimuli were presented at opposite polarities (180°phase shifted) for each test condition and the corresponding AEPs were averaged by the BioSig RP software in order to eliminate stimulus artefacts. The sound pressure level (SPL) of tone-burst stimuli was reduced in 4 dB steps until the AEP waveform was no longer apparent. The lowest SPL for which a repeatable AEP trace could be obtained, which was determined by overlaying replicate traces, was considered the threshold [56], [66]. A hydrophone (Brüel & Kjaer 8101, Naerum, Denmark; frequency range 1 Hz to 80 kHz±2 dB, voltage sensivity – 184 dB re 1 VµPa^−1^) was positioned near the right side of each fish (2 cm away) to determine absolute SPLs values underwater, close to the subjects.

### Temporal resolution measurements

In order to analyze the temporal resolution ability at different temperature, the technique described by Wysocki and Ladich [54] was applied. Single clicks and double-clicks were generated using TDT System II and TDT ‘SigGen’ software and fed through a DA1 digital-analog converter, a PA4 programmable attenuator, and a power amplifier (Denon PMA 715R) to the air speaker (Tannoy System 600). Each type of stimulus (single click and double-click) was presented to the animals at a repetition rate of 35 s^−1^. Double-click stimuli were presented at 28 dB above hearing threshold. Ten different click periods were presented, beginning with the shortest click period. Click periods tested were 0.3, 0.5, 1, 1.5, 2, 2.5, 3, 3.5, 4 and 5 ms.

The amplitudes of the responses to the second click of each pair of clicks were measured and compared to the response to a single click following the method used in Wysocki and Ladich (2002). The most consecutive peaks were used for analysis. The AEP components were denominated with P for positive peaks (directed upwards) and N for negative peaks (directed downwards) by ascending numbers. The main peaks for analysis were N1, N2, P2 and P3. First, the hearing threshold in response to a single click was determined, followed by a presentation of double-clicks at 28 dB above hearing threshold.

A point-to-point subtraction operation was conducted [54] to isolate the response to the second click within a pair of clicks. The AEP in response to a single click was substracted from the response to a double-click. The shortest click period at which a second response was still detectable was classified as the minimum resolvable click period.

### Latency measurements

The latency was defined as the time between the onset of the single click stimulus and the first four constant peaks of the AEP recorded in responses to this click stimulus. The most constant peaks in the AEPs were N1, P1, N2 and P2 (see Fig. 2 in [54]). The single click was presented at 28 dB above hearing threshold.

### Statistical analyses

All data were tested for normal distribution using the Kolmogorov-Smirnov-test and when data were normally distributed, parametric statistical tests were applied. Stridulation sounds data determined at three different experimental temperatures were compared using a non-parametric test (Friedman-test followed by a Wilcoxon-test). A Kruskal-Wallis test was applied to calculate differences in drumming sound characteristics because only five individuals produced drumming sounds at all temperatures. Audiograms obtained at the three temperatures (22°C, 30°C and 22°C repeated) were compared by a two-factorial analysis of variance (ANOVA) using a general linear model where one factor was temperature and the other was frequency. The temperature factor alone should indicate overall differences in sensitivity between temperatures and in combination with the frequency factor if different tendencies exist at different frequencies of the audiograms. A Post-hoc test (Bonferroni) revealed differences between temperatures. All statistical tests were run using SPSS 17.0. The significance level was set at p≤0.05.
